# Empagliflozin attenuates intestinal inflammation through suppression of nitric oxide synthesis and myeloperoxidase activity in in vitro and in vivo models of colitis

**DOI:** 10.1007/s10787-023-01227-8

**Published:** 2023-04-22

**Authors:** Adam Makaro, Mikołaj Świerczyński, Kacper Pokora, Barbara Sarniak, Radzisław Kordek, Jakub Fichna, Maciej Salaga

**Affiliations:** 1 Department of Biochemistry, Faculty of Medicine, Medical University of Lodz, Lodz, Poland; 2 Department of Pathology, Faculty of Medicine, Medical University of Lodz, Lodz, Poland

**Keywords:** Inflammatory bowel diseases, Empagliflozin, SGLT-2, DSS-induced colitis

## Abstract

Inflammatory bowel diseases (IBD) are characterized by chronic and relapsing inflammation affecting the gastrointestinal (GI) tract. The incidence and prevalence of IBD are relatively high and still increasing. Additionally, current therapeutic strategies for IBD are not optimal. These facts urge todays’ medicine to find a novel way to treat IBD. Here, we focused on the group of anti-diabetic drugs called gliflozins, which inhibit sodium glucose co-transporter type 2 (SGLT-2). Numerous studies demonstrated that gliflozins exhibit pleiotropic effect, including anti-inflammatory properties. In this study, we tested the effect of three gliflozins; empagliflozin (EMPA), dapagliflozin (DAPA), and canagliflozin (CANA) in in vitro and in vivo models of intestinal inflammation. Our in vitro experiments revealed that EMPA and DAPA suppress the production of nitric oxide in LPS-treated murine RAW264.7 macrophages. In in vivo part of our study, we showed that EMPA alleviates acute DSS-induced colitis in mice. Treatment with EMPA reduced macro- and microscopic colonic damage, as well as partially prevented from decrease in tight junction gene expression. Moreover, EMPA attenuated biochemical inflammatory parameters including reduced activity of myeloperoxidase. We showed that SGLT-2 inhibitors act as anti-inflammatory agents independently from their hypoglycemic effects. Our observations suggest that gliflozins alleviate inflammation through their potent effects on innate immune cells.

## Introduction

Inflammatory bowel diseases (IBD), including ulcerative colitis (UC) and Crohn’s disease (CD), are characterized by chronic and relapsing inflammation affecting the gastrointestinal (GI) tract. IBD shares common traits, such as dysbiosis and similar profile of overactive pro-inflammatory cytokines, with other diseases. One of them is type 2 diabetes mellitus (DM) (Jurjus et al. 2016).

The incidence and prevalence of IBD are high and still increasing. In Europe, approximately 2.2 million people suffer from CD or UC (Ananthakrishnan 2015). As a result, healthcare systems experience high direct costs associated with consultations, diagnostic procedures and drug therapies. Additionally, IBD sufferers incur indirect costs related to workplace productivity losses. Current therapeutic strategies for IBD are based on corticosteroids, 5-aminosalicylic acid and biologics. However, even treatment with the use of anti-tumor necrosis factor alpha (TNFα) antibodies, considered one of the most effective, poses risk of serious adverse effects or development of tolerance (Strik et al. 2016). Thus, the target of todays’ medicine is to find a novel way to treat IBD.

One of the newest anti-diabetic drugs are sodium glucose co-transporter type 2 (SGLT-2) competitive inhibitors called gliflozins. The US Food & Drug Administration (FDA) approved three medications of this class: empagliflozin (EMPA) dapagliflozin (DAPA) and canagliflozin (CANA). SGLT-2 is found in many tissues but is primarily expressed in proximal tubules of the kidney and its inhibition leads to glycosuria. Numerous studies demonstrated that gliflozins exhibit pleiotropic effect including cardio- and renoprotective properties (Yamada et al. 2021). Moreover, pre-clinical research showed that gliflozins also exert anti-inflammatory effect regardless of its hypoglycemic activity. For example, SGLT-2 inhibition significantly attenuated inflammatory responses in vitro, which was demonstrated mainly in macrophages treated with lipopolysaccharide (LPS). EMPA decreased the production of inflammatory cytokines: TNFα, interleukin (IL) 1β, and IL-6 in LPS-stimulated RAW264.7 cells (Lee et al. 2021; Liu et al. 2021; Xu et al. 2018). Moreover, EMPA inhibited the production of other molecules playing key roles in inflammation, e.g. prostaglandin E2 (PGE2), cyclooxygenase-2 (COX-2) and inducible nitric oxide synthase (iNOS) in the same experimental model (Lee et al. 2021). Moreover, in RAW264.7 macrophages and human umbilical vein endothelial cells (HUVECs), DAPA decreased the levels of inflammatory cytokines and increased the expression of anti-inflammatory miR-146a. This effect of DAPA was observed in LPS-treated cells exposed to both normal and high glucose levels (Abdollahi et al. 2022). In another study, CANA decreased the levels of TNFα, IL-1α, IL-6 and reactive oxygen species (ROS) in LPS-stimulated RAW264.7 macrophages, as well as TNFα and IL-1β in LPS-treated human leukemia monocytic cells (THP-1) (Xu et al. 2018).

Rodent studies also showed anti-inflammatory effects of SGLT-2 inhibitors. EMPA significantly decreased the concentrations of serum IL-1β, IL-6 and IL-10 in non-diabetic mice (Liu et al. 2021). In another study, CANA reduced the levels of TNFα and IL-6 in LPS-treated mice (Xu et al. 2018). Moreover the use of gliflozins decreased biochemical parameters of inflammation and reduced macro- and microscopic colonic damage in rat model of acetic-acid colitis (El-Rous et al. 2021; Morsy et al. 2021; Zaghloul et al. 2022). Similarly, dextran sulfate sodium (DSS)-induced colonic inflammation in mice was reduced by EMPA alone or in combination with metformin (Youssef et al. 2021).

Hattori et al. showed that patients receiving EMPA had high sensitivity C-reactive protein (CRP) reduced by 54% after 12 months of treatment (Hattori 2018). Another human study showed greater anti-inflammatory effects of CANA than glimepiride in patients with type 2 DM (Heerspink et al. 2019).

Results of all mentioned studies suggest that gliflozins possess anti-inflammatory activity but to date its mechanism was not fully characterized. Therefore, the aim of this project was to verify the anti-inflammatory effect of gliflozins, which is suggested by numerous studies as partly independent from its hypoglycemic activity and to investigate into biomolecular changes in cell lines and the GI tract that accompany treatment with those drugs For this purpose, we evaluated the effects of gliflozins in in vitro and in vivo models of GI inflammation. First, we evaluated the influence of EMPA, DAPA and CANA on the inflammatory responses in RAW264.7 macrophages and Caco-2 cells. Then, we assessed the effect of selected gliflozin, EMPA, on GI inflammation in vivo. For this purpose, we used two models of DSS–induced colitis and measured the macro-/microscopic and biochemical parameters of inflammation in the mouse colon.

## Materials and methods

### Cell line culture

The RAW264.7 mouse macrophages (ATCC: TIB-71) were cultured in Dulbecco’s Modified Eagle Medium (Gibco) supplemented with 10% bovine calf serum (BCS), 2 mM Alanine-Glutamine, 0.5% penicillin–streptomycin (P/S), 1 mM sodium pyruvate, and 25 mM HEPES. The cells were grown in a humidified atmosphere of 5% CO_2_ at 37 ℃. The medium was refreshed every two or three days and the cells were passaged after reaching approx. 80% confluence.

The Caco-2 human colonic epithelial cells (ATCC: HTB-37) were cultured in Eagle’s Minimal Essential Medium (Gibco) with addition of 20% fetal bovine serum (FBS), 4 mM Ala-Gln, 0.1 mM nonessential amino acids, 0.5% P/S, 1 mM sodium pyruvate, and 25 mM HEPES. The cells were grown in a humidified atmosphere of 5% CO_2_ at 37 ℃. The cells were trypsinized and passaged after reaching approx. 80% confluence.

### Cytotoxicity assessment

The effect of gliflozins on the cell viability of RAW264.7 macrophages was evaluated with the use of neutral-red uptake (NRU) assay. Gliflozins were tested in following concentrations: 10, 20, 40, 80 µM (EMPA), 40, 80, 100, 200 µM (DAPA), 20, 40, 80, 100 µM (CANA) and budesonide was applied at the concentration of 10 µM as a positive control (Salaga et al. 2021).

The assay is based on the staining of living cells by neutral red (NR), which indicates the lysosomal activity of viable cells. The cells were seeded on 96-well plates (20,000 cells/well) and exposed to the compounds for 48 h before the measurement of cytotoxicity. Then, the medium was removed and 100 µl/well of 0.05 mg/ml NR solution in culture medium was added. After 1 h incubation, cells were washed with phosphate buffer saline (PBS, pH 7.4) and 100 µl/well of 40% ethanol, and 10% acetic acid in water was used to dissolve the dye. The plates were shaken for 10 min and the absorbance was measured at 540 nm in a microplate reader (iMARK Microplate Reader, Biorad, Hertfordshire, UK). Cytotoxicity was expressed as a percentage of cells without any treatment.

### Griess assay

The effect of gliflozins on nitrite secretion of RAW264.7 cells was evaluated with Griess Assay. The cells were seeded on 96-well plates (20,000 cells/well) and incubated with standard culture medium (control) or medium with 0.5 µg/ml LPS with or without gliflozins or budesonide. After 24 h of incubation medium was removed, and solutions containing gliflozins or budesonide, without LPS, were added. After another 24 h, 100 µl of cell culture supernatant was mixed with 100 µl of Griess reagent water solution (40 mg/ml), and the mixture was incubated in the dark for 15 min. Next, the absorbance was read at 540 nm. Nitrite concentration was expressed as a percentage of the cells treated with LPS only.

### In vitro model of LPS-induced inflammation

The effect of gliflozins on mRNA expression of proinflammatory cytokines in RAW264.7 cells was evaluated after stimulation with LPS. The cells were seeded on 6-well plates (6,000,000 cells/well) and incubated with standard culture medium (control) or medium with 0.5 µg/ml LPS + DMSO (0.004% for EMPA, 0.01% for DAPA and CANA) with or without gliflozins. After 24 h of incubation medium was removed, and solutions containing gliflozins, without LPS, were added. After another 24 h, total RNA was isolated from the cells. Furthermore, mRNA expression of TNFα, IL-1β, IL-6 and IL-10 was measured by using qPCR.

### In vitro model of cytokine and LPS-induced inflammation

The effect of gliflozins on inflammatory response of Caco-2 cells was evaluated with the model proposed by Van De Walle et al. (2010). The cells were seeded on a 24-well plate (100,000 cells/well) and incubated for 24 h with standard culture medium. Then, a mixture of TNFα (50 ng/ml), Il-1β (25 ng/ml), interferon gamma (IFNγ; 50 ng/ml), and LPS (100 ng/ml) was added to each well. Experimental groups were additionally treated with EMPA and DAPA in concentrations selected based on the out-comes of cytotoxicity assessment: EMPA; 40 µM, and DAPA; 100 µM (0.25% DMSO). After 12 h of incubation, cell culture medium was harvested and used for IL-6 measurement with a human IL-6 ELISA kit (cat. no. 950.030.096, Diaclone, France) according to manufacturer instructions. Outcomes were expressed as a percentage of cells treated with a mixture of cytokines and LPS only.

### Animals

We used experimentally naive male Balb/c mice (Animal House at the University of Lodz, Poland) weighing 20–25 g. Animals were housed at a constant temperature (22 °C) and maintained under a 12-h light/dark cycle in sawdust-lined plastic cages. Chow pellets and tap water were provided ad libitum. All animal protocols were approved by the Medical University of Lodz Animal Care Committee (Protocol 38/ŁB177/2020) and complied with the European Communities Council Directive of 22 September 2010 the EU (2010/63/EU). All efforts were made to minimize animal suffering and to reduce the number of animals used. Groups of 10 (control groups) or 12 animals (experimental groups) were used in all in vivo experiments.

### Acute and chronic-relapsing models of DSS-induced colitis

Acute colitis was induced by the addition of DSS to drinking water from day 0 to day 4 (3% wt/vol; molecular weight 40,000; MP Biomedicals, Aurora, OH, Lot No. 5237 K). On days 5 to 7, animals received water without DSS. Control animals were receiving tap water throughout the whole experiment (Chassaing et al. 2014; Salaga et al. 2021).

Chronic-relapsing colitis was induced by 3 cycles of 5 days treatment with DSS (2% wt/vol; molecular weight 40,000; MP Biomedicals, Aurora, OH, Lot No. 5237 K) in drinking water followed by 4 days of water without DSS (3 cycles, 9 day each, 27 days in total). Control animals were receiving tap water throughout the whole experiment (Chassaing et al. 2014).

### Pharmacological treatment

In acute model of colitis, 5% DMSO solutions of EMPA were orally administered at the doses 1 mg/kg bw or 5 mg/kg bw once daily (on days 3–6). 5% DMSO was also administered to control and DSS-treated groups.

In chronic-relapsing model, 5% DMSO solutions of EMPA were orally administered at the doses 0.3 mg/kg bw or 1 mg/kg bw once daily (on days 10–27). 5% DMSO was also administered to control and DSS-treated groups.

### Evaluation of colonic damage

On day 7 (acute) and 28 (chronic) all mice were sacrificed by cervical dislocation, and the macroscopic evaluation was performed by two researchers (AM and MSw). The entire colon was excised and weighed with fecal content. Then, the colon was opened longitudinally and washed. A total macroscopic damage score was expressed in arbitrary units (A.U.) calculated based on the following parameters: colon epithelial damage determined by a number of ulcers (0–3), stool score (where 0 means normal, well-shaped fecal pellets and 3 means diarrhea), colon weight and length scores (0–4) calculated as a loss of each parameter in relation to the control group (0 points, ≤ 5% change; 1 point, 5–14% change; 2 points, 15–24% change; 3 points, 25–35% change; and 4 points, ≥ 35% change). The presence (score = 1) or absence (score = 0) of fecal blood was also recorded. The macroscopic scoring was performed in a blind manner (Salaga et al. 2017).

### Determination of tissue myeloperoxidase (MPO) activity

One-centimeter segments of colon were weighed and homogenized in hexa-decyltrimethylammonium bromide (HTAB) buffer (0.5% HTAB in 50 mM potassium phosphate buffer, pH 6.0; 50 mg of tissue/ml) immediately after isolation and the homogenate was centrifuged (15 min, 13,200 × g, 4 °C). Then, 7 µl of supernatants were added to each well on a 96-well plate containing 200 µl of 500 mM potassium phosphate buffer supplemented with 0.167 mg/ml of O-dianisidine hydrochloride and 0.05 µl of 1% H_2_O_2_. Absorbance was measured at 450 nm (iMARK Microplate Reader, Biorad, United Kingdom). All measurements were performed in triplicate. MPO was ex-pressed in milliunits per gram of wet tissue, 1 unit being the quantity of enzyme able to convert 1 µmol of H_2_O_2_ to water in 1 min at room temperature. Units of MPO activity per 1 min were calculated from a standard curve using purified peroxidase enzyme.

### Histopathological evaluation

After the macroscopic damage evaluation, segments of the distal colon were stapled flat, mucosal side up, onto cardboard and fixed in 10% neutral-buffered formalin for 24 h at 4 °C. Samples were then dehydrated in sucrose, embedded in paraffin, sectioned at 5 μm and mounted onto slides. Subsequently, sections were stained with hematoxylin and eosin and examined using an Axio Imager A2 microscope (Carl Zeiss, Oberkochen, Germany). Photographs were taken using a digital imaging system consisting of a digital camera (Axiocam 506 clolor, Carl Zeiss, Germany) and image analysis software (Zen 2.5 blue edition, Carl Zeiss, Germany). A total microscopic damage score was expressed in arbitrary units (A.U) calculated based on the following parameters assessed by two researchers (AM and KP): the presence (score = 1) or absence (score = 0) of goblet cell depletion, the presence (score = 1) or absence (score = 0) of crypt abscesses, the destruction of mucosal architecture (normal = 1, moderate = 2, extensive = 3), the extent of muscle thickening (normal = 1, moderate = 2, extensive = 3), and the presence and degree of cellular infiltration (normal = 1, moderate = 2, transmural = 3) (Salaga et al. 2017).

### RNA isolation, reverse transcription and qPCR

Briefly, total RNA was isolated from RAW264.7 cells (in vitro) or from the distal sections of large intestine (weighing 20–30 mg) from both healthy and DSS-treated animals (in vivo), in accordance with the manufacturer’sprotocol using Total RNA Mini Plus kit (A&A Biotechnology, Gdansk, Poland). RNA was eluted from ion ex-change columns by diethyl pyrocarbonate (DEPC)-treatedwater (40 µl). The purity and quantity of isolated RNA was estimated using Colibri Microvolume Spectrophotome-ter (Biocompare, San Francisco, CA, USA). Total RNA (in vitro: 0.1 µg, in vivo: 0.5 µg) was transcribed to cDNA with Maxima First Strand cDNA Synthesis Kit for RT-qPCR (Thermo Fisher Scientific, Waltham, MA, USA) in accordance with the manufacturer’s protocol. Quantitative assay of the expression was executed using fluorescently labeled probes (Life Technologies, Carlsbad, CA, USA): TNFα (Mm00443258_m1), Il-1β (Mm00434228_m1), Il-6 (Mm00446190_m1), claudin 2 (CLDN-2) (Mm00516703_s1), CLDN-3 (Mm00515499_s1), CLDN-4 (Mm00515514_s1), CLDN-7 (Mm00516817_m1), CLDN-10 (Mm01226326_g1) and HPRT (Mm03024075_m1) as endogenous control on Lightcycler 96 (Roche, Basel, Switzerland) using Takyon One-Step Kit Converter (Euragentec, Seraing, Belgium) according to the manufacturer’s protocol. All experiments were conducted in duplicate. The threshold cycle (Ct) values for studied genes were normalized to Ct values received for HPRT. The relative quantity of mRNA copies was calculated using the equation: 2-ΔCt × 1000. Then all values were normalized and expressed as a fold-change relative to the control.

### Statistics

The statistical analysis was performed using Prism 8.2.1 (GraphPad Software Inc., La Jolla, CA, USA) with the use of one-way ANOVA followed by the Dunnett’s test for multiple comparisons. The data are expressed as the means ± SEM.

## Results

### Effect of gliflozins on cellular viability, LPS-stimulated NO production and mRNA ex-pression of TNFα, IL-1β, IL-6 and IL-10 in RAW264.7 macrophages

**Fig. 1:**
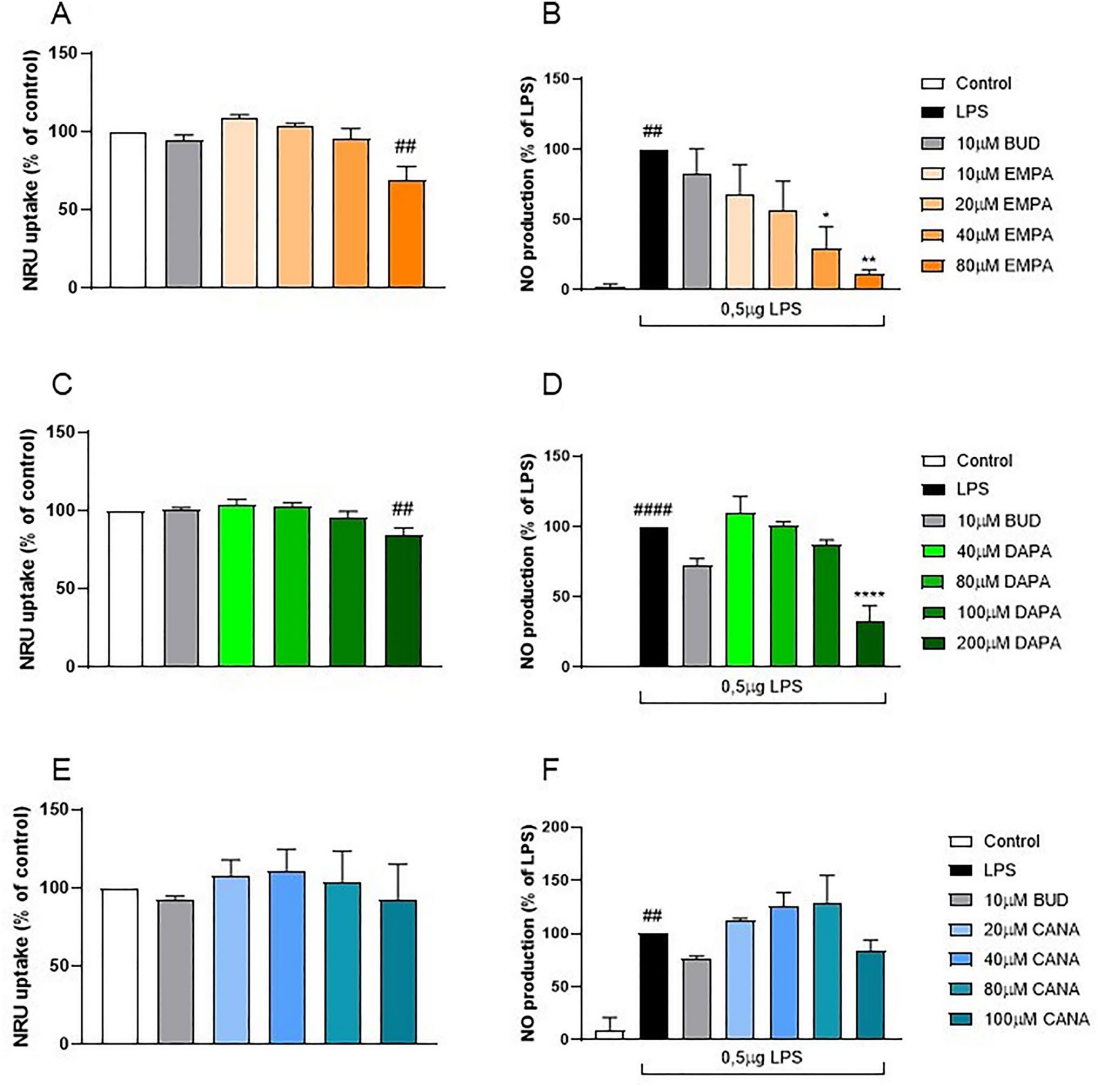
Effect of gliflozins on survival of RAW264.7 macrophages. The viability of cells was determined by neutral-red (NRU) uptake after treatment with various concentrations of EMPA (**A**), DAPA (**C**), and CANA (**E**). Effect of gliflozins on nitric oxide (NO) secretion of RAW264.7 cells after 24 h stimulation with lipopolysaccharide (LPS), and treatment with various concentrations of respective gliflozins (**B**, **D**, **F**). Budesonide (BUD, 10 µM) was used for comparison in each experiment. Values are mean ± SEM of 3 experiments, 6 replicates each. Significance of differences between means: ^##^p < 0.01, ^####^p < 0.0001 versus control cells, *p < 0.05, **p < 0.01, ****p < 0.0001 versus LPS-treated group

Initially we measured the expression of SGLT-2 in RAW 264.7 and Caco-2 cells by Western blotting analysis (data not shown) and observed that this proteins is present in those cell lines. Then, in order to assess the cytotoxicity and anti-inflammatory activity of EMPA, DAPA and CANA we used NRU and Griess assays in RAW 264.7 cells. We did not observe a toxic effect of EMPA at the concentrations of 10, 20, and 40 µM. However, EMPA at 80 µM displayed significant cytotoxicity (Fig. 1a). Among the tested concentrations of DAPA (40, 80, 100, 200 µM), only the highest one induced slight but significant reduction of the cell viability (Fig. 1c). NRU assay did not show cytotoxicity of CANA at any of following concentrations: 20, 40, 80, 100 µM (Fig. 1e). However, we observed high cytotoxicity of CANA at 200 µM (data not shown). We also evaluated the effect of gliflozins on the inflammatory response in RAW264.7 cells. We observed that treatment with EMPA resulted in a dose-dependent decrease in the level of NO production in LPS-stimulated macrophages. At concentrations of 10, 20, 40, and 80 µM, EMPA reduced the NO secretion by 32.33%, 43.73%, 70.43% (p = 0.0195) and 88.47% (p = 0.0037), respectively (Fig. 1b). The treatment with DAPA at 200 µM inhibited the production of NO by 67.6% (p < 0.0001) (Fig. 1d). Significant inhibition of NO synthesis was not apparent in macrophages exposed to CANA (Fig. 1e). Positive controls were treated with budesonide at 10 µM in all experiments.

**Fig. 2:**
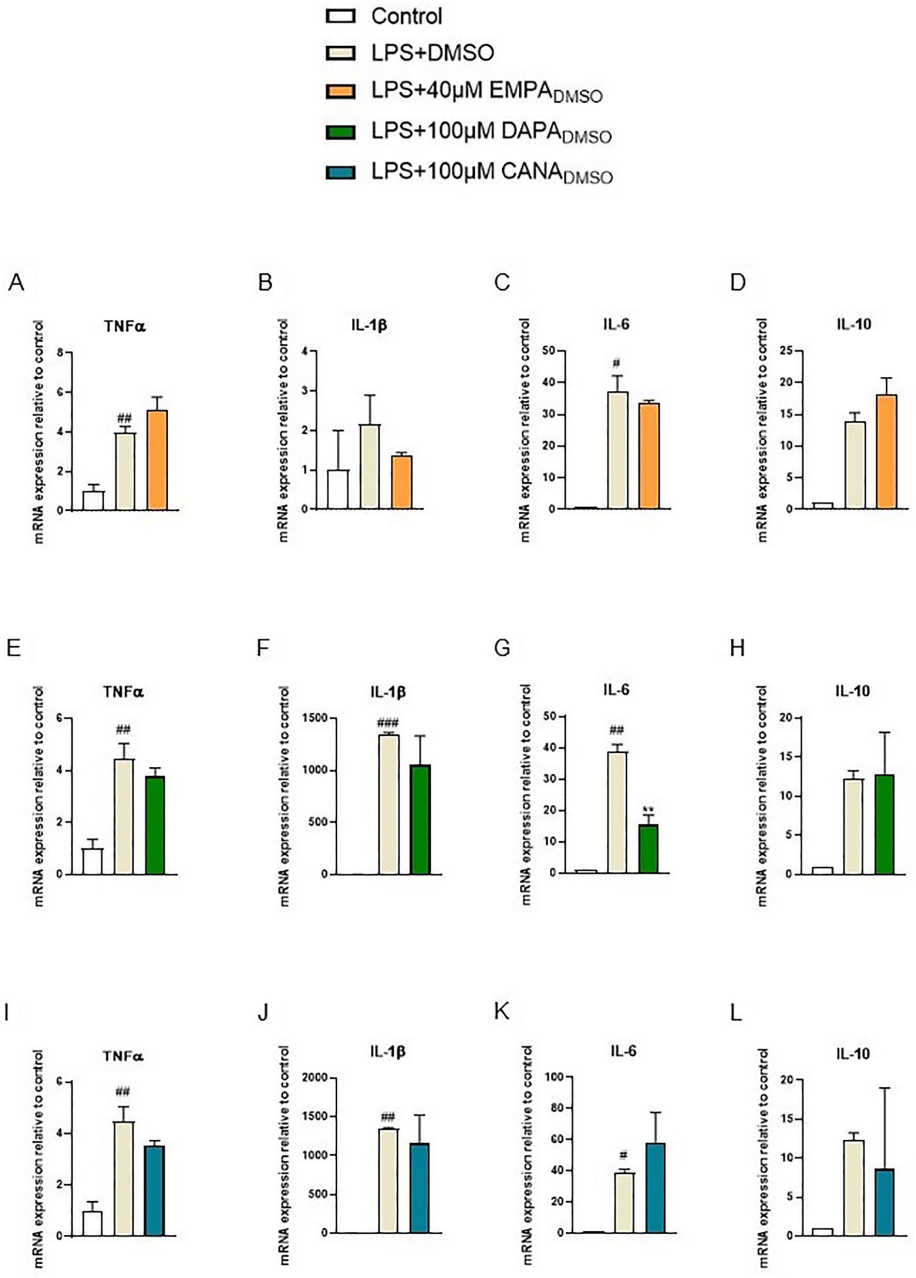
Effect of 40 µM of EMPA (**A**–**D**), 100 µM of DAPA (**E**–**H**), and 100 µM of CANA (**I**–**L**) on mRNA expression of proinflammatory cytokines in RAW264.7 cells after 24 h stimulation with lipopolysaccharide (LPS). Figure shows data for TNFα (**A**, **E**, **I**), IL-1β (**B**, **F**, **J**), IL-6 (**C**, **G**, **K**), and IL-10 (**D**, **H**, **L**) presented in relative units compared to control. LPS with DMSO (0.004% for EMPA, 0.01% for DAPA and CANA) was used for comparison as positive control. Values are mean ± SEM, of 3 replicates. Significance of differences between means: ^#^p < 0.05, ^##^p < 0.01, ^###^p < 0.001 versus control group, **p < 0.01 versus LPS-treated group

In the next step, we evaluated the effects of gliflozins on the levels of mRNA ex-pression of cytokines in LPS-treated RAW264.7 cells. For this purpose, we tested the highest non-toxic concentrations of drugs; 40 µM for EMPA and 100 µM for DAPA and CANA. We observed that LPS significantly increased mRNA expression of TNFα, IL-1β, and IL-6 (Fig. 2a–k). DAPA at the concentration of 100 µM significantly attenuated this effect for IL-6 (p = 0.0028) (Fig. 2g), and partially reduced expression of TNFα and IL-1β (Fig. 2e, f). Moreover, 40 µM of EMPA partially decreased expression of IL-1β and IL-6 (Fig. 2b, c). Similarly, 100 µM of CANA partially reduced expression of TNFα and IL-1β (Fig. 2i, j).

In summary, EMPA and DAPA significantly alleviated inflammatory response in RAW264.7 cells. Therefore, we focused on these gliflozins in further in vitro experiments. We selected following concentrations; 40 µM for EMPA and 100 µM for DAPA for subsequent studies.

### Effect of EMPA and DAPA on inflammatory response in cytokine and LPS-stimulated Caco-2 cells

**Fig. 3:**
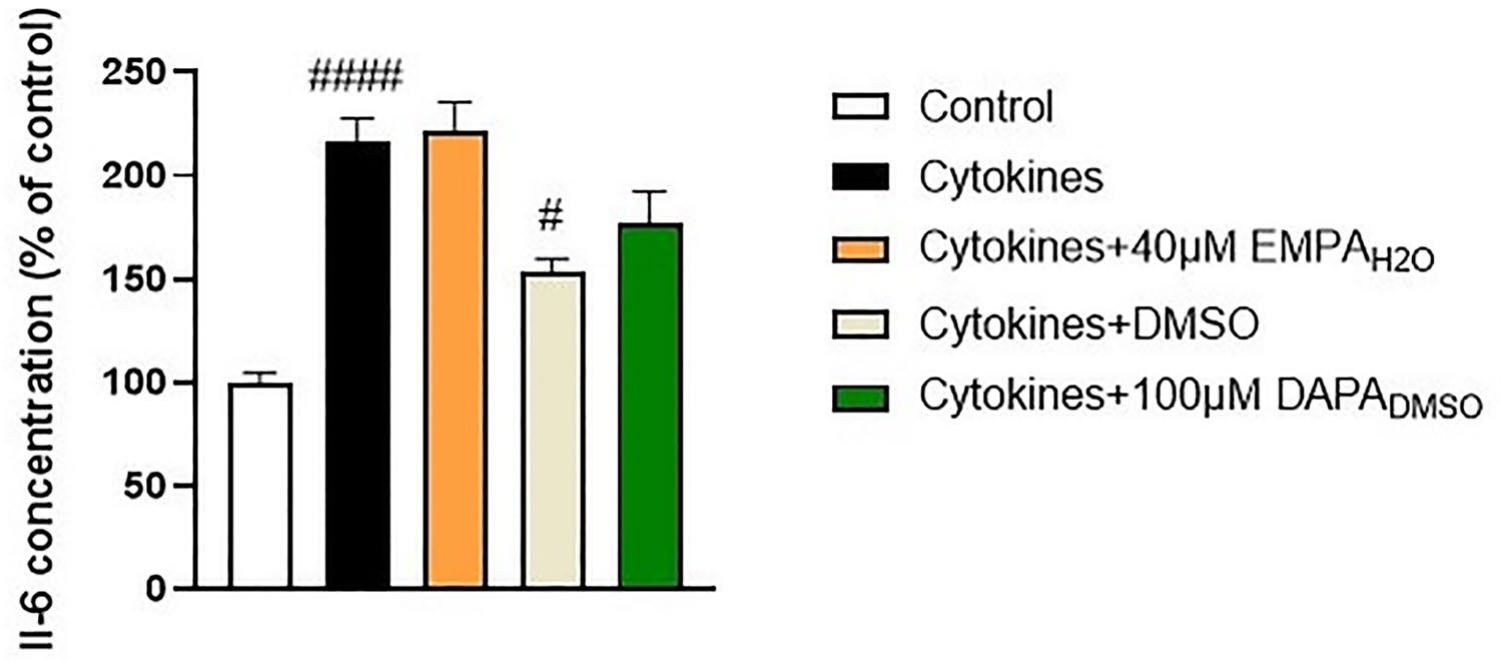
Effect of EMPA and DAPA on IL-6 secretion in Caco-2 cells after 12 h stimulation with the cocktail of TNFα, IL-1β, IFNγ and LPS. Values are mean ± SEM of 4 replicates. Significance of differences between means: ^#^p < 0.05, ^####^p < 0.0001 versus control cells

In the following step of in vitro experiments, we measured the secretion of IL-6 in cytokine (TNFα, IL-1β, IFNγ) and LPS-stimulated Caco-2 cells. We treated the cells with water solution of EMPA (40 µM), and 0.25% DMSO solution of DAPA (100 µM). DMSO inhibited the inflammatory response in Caco-2 cells, thus we used 0.25% DMSO–treated group as a control for cells exposed to DAPA (Hollebeeck et al. 2011). As a result, treatment with both gliflozins did not influence the inflammatory response in Caco-2 cells (Fig. 3).

### Effect of EMPA on the acute model of DSS-induced colitis in mice

The results of the in vitro part of our study allowed us to choose EMPA as the best candidate to evaluate the anti-inflammatory effect of gliflozins in the mouse GI tract. For this purpose, we used well-established mouse models of colitis induced by DSS. Firstly, we tested the drug in acute model of colitis induced by the addition of 3% DSS to drinking water from day 0 to day 4. EMPA was orally administered at two doses: 1 mg/kg or 5 mg/kg once daily from day 3 to day 6.

**Fig. 4:**
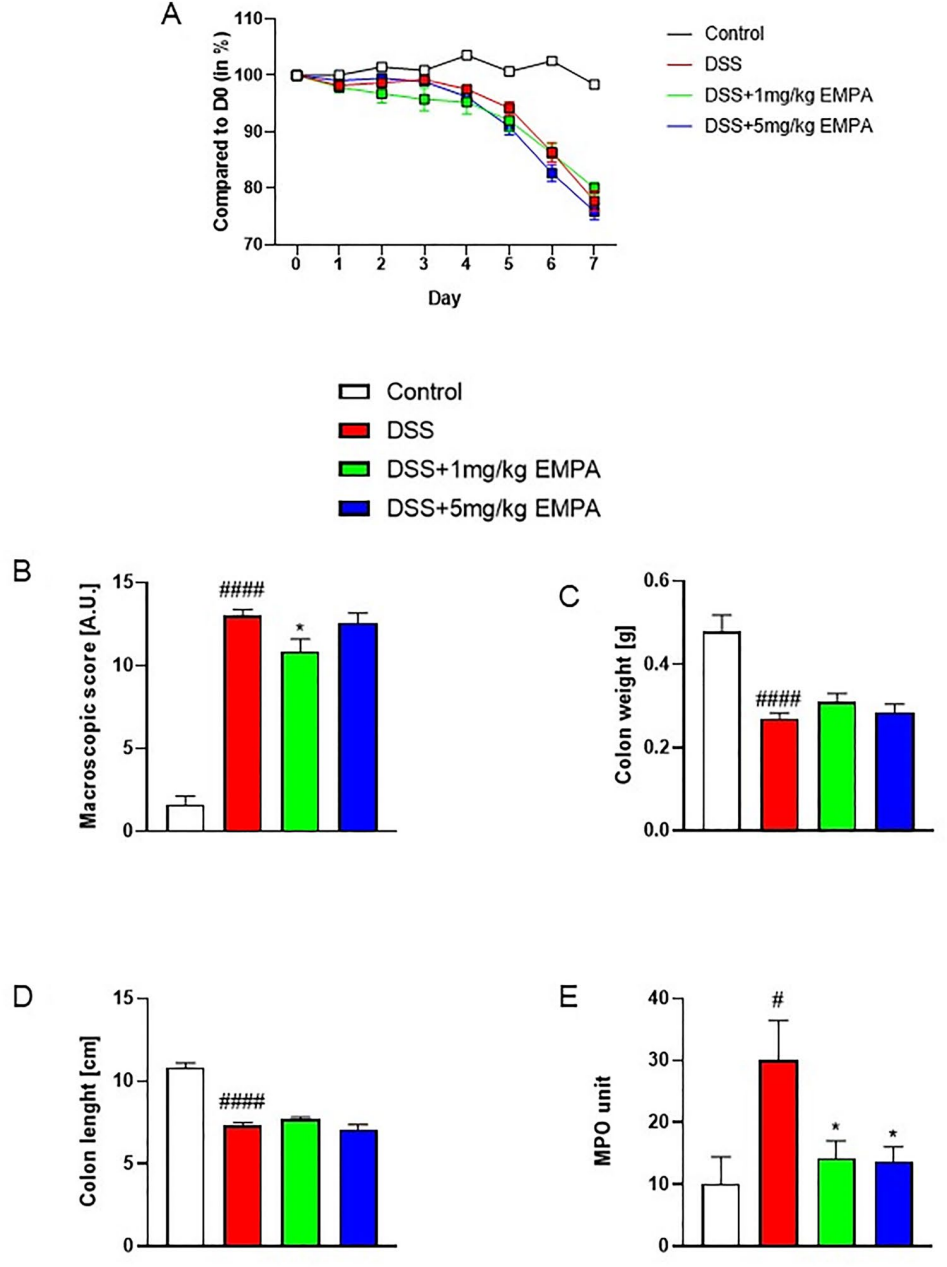
Effect of EMPA on parameters of acute inflammation in DSS-treated mice. EMPA was orally administered at two doses: 1 mg/kg or 5 mg/kg once daily (on days 3–6). Figure shows data for body weight (**A**), macroscopic score (**B**), colon weight (**C**), colon length (**D**), and MPO activity (**E**). Values are mean ± SEM, of 9–11 mice per group. Significance of differences between means: ^#^p < 0.05, ^####^p < 0.0001 versus control group, *p < 0.05 versus DSS-treated group. A.U. arbitrary unit

Mice exposed to DSS developed severe colonic injury, manifested by body weight loss, intensified macroscopic damage, decreased colon weight and colon length (Fig. 4a–d). The macroscopic damage score (13 ± 0.36 for DSS vs 1.6 ± 0.54 for control, p < 0.0001) was reduced in animals treated with 1 mg/kg EMPA (10.83 ± 0.76, p = 0.032) (Fig. 4b). DSS-induced colitis was characterized by increased MPO activity (30.09 ± 6.38U for DSS vs 10.11 ± 4.34U for control, p = 0.012), which was significantly inhibited by EMPA (14.29 ± 2.76U, p = 0.038 for 1 mg/kg; 13.69 ± 2.41U, p = 0.036 for 5 mg/kg) (Fig. 4e).

**Fig. 5:**
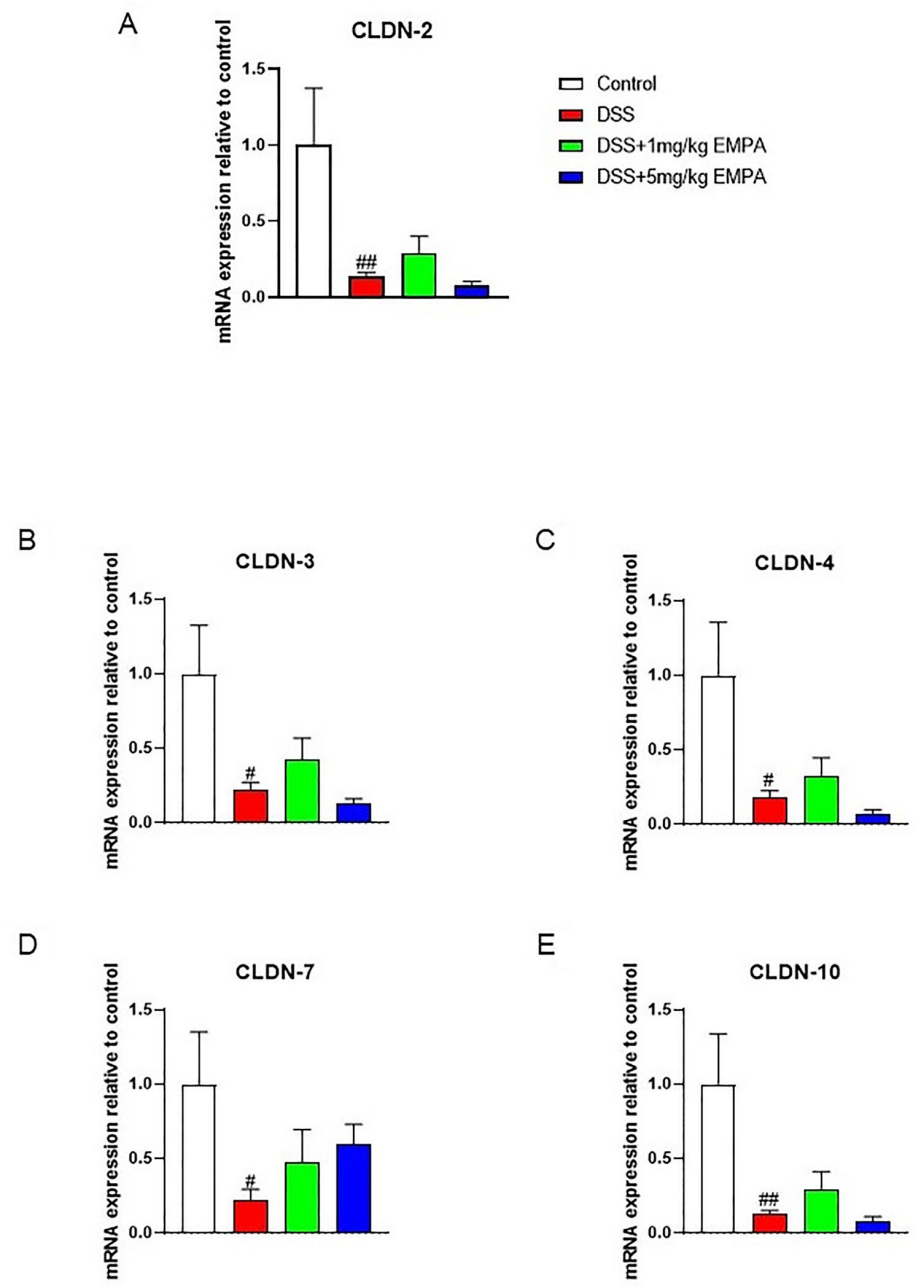
Effect of EMPA on the expression of TJPs in the colon of mice with acute DSS-induced colitis. EMPA were orally administered at two doses: 1 mg/kg or 5 mg/kg once daily (on days 3–6). Figure shows data for CLDN-2 (**A**), CLDN-3 (**B**), CLDN-4 (**C**), CLDN-7 (**D**), and CLDN-10 (**E**) presented in relative units compared to control. Values are mean ± SEM, of 9–11 mice per group. Significance of differences between means: ^#^p < 0.05, ^##^p < 0.01 versus control group

**Fig. 6:**
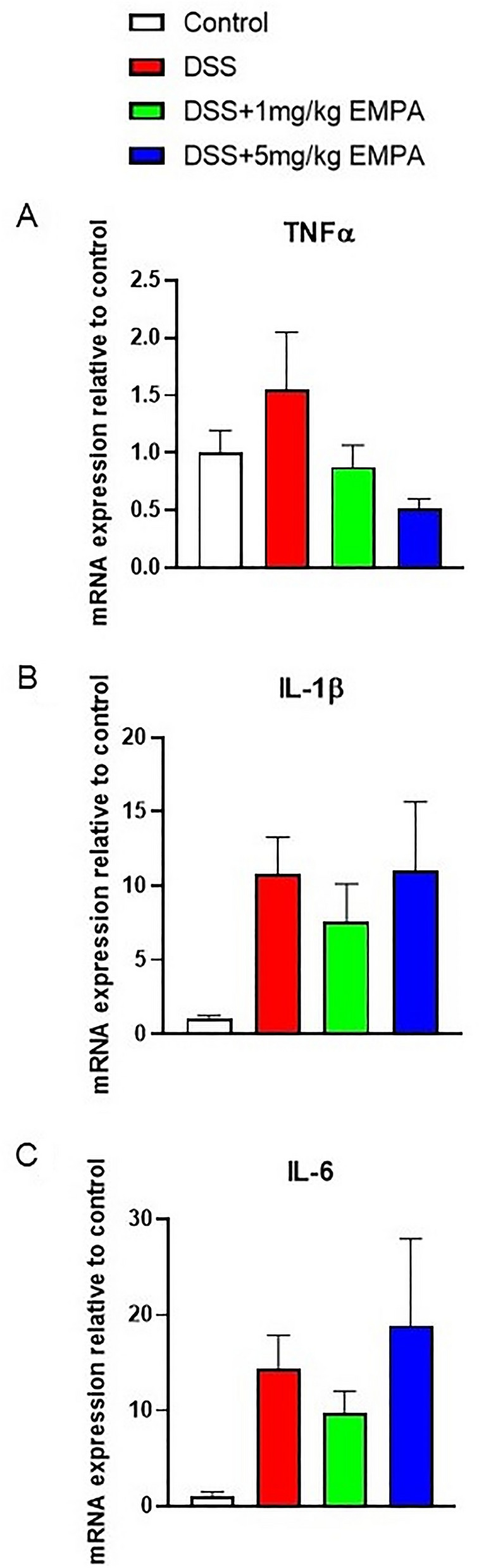
Effect of EMPA on pro-inflammatory cytokines gene expression in the colon of mice with acute DSS-induced colitis. EMPA were orally administered at two doses: 1 mg/kg or 5 mg/kg once daily (on days 3–6). Figure shows data for TNFα (**A**), IL-1β (**B**), and IL-6 (**C**) presented in relative units compared to control. Values are mean ± SEM, of 9–11 mice per group

We observed that treatment with DSS significantly decreased the expression of tight junction proteins (TJPs) mRNA, including CLDN-2 (p = 0.0095), CLDN-3 (p = 0.0012), CLDN-4 (p = 0.011), CLDN-7 (p = 0.0038), and CLDN-10 (p = 0.0047) (Fig. 5a–e). Moreover, DSS treatment resulted in increased expression of inflammatory cytokines (TNFα, IL-1β, IL-6), (Fig. 6a–c). These effects of DSS were partially, but not significantly, attenuated by 1 mg/kg of EMPA.

**Fig. 7:**
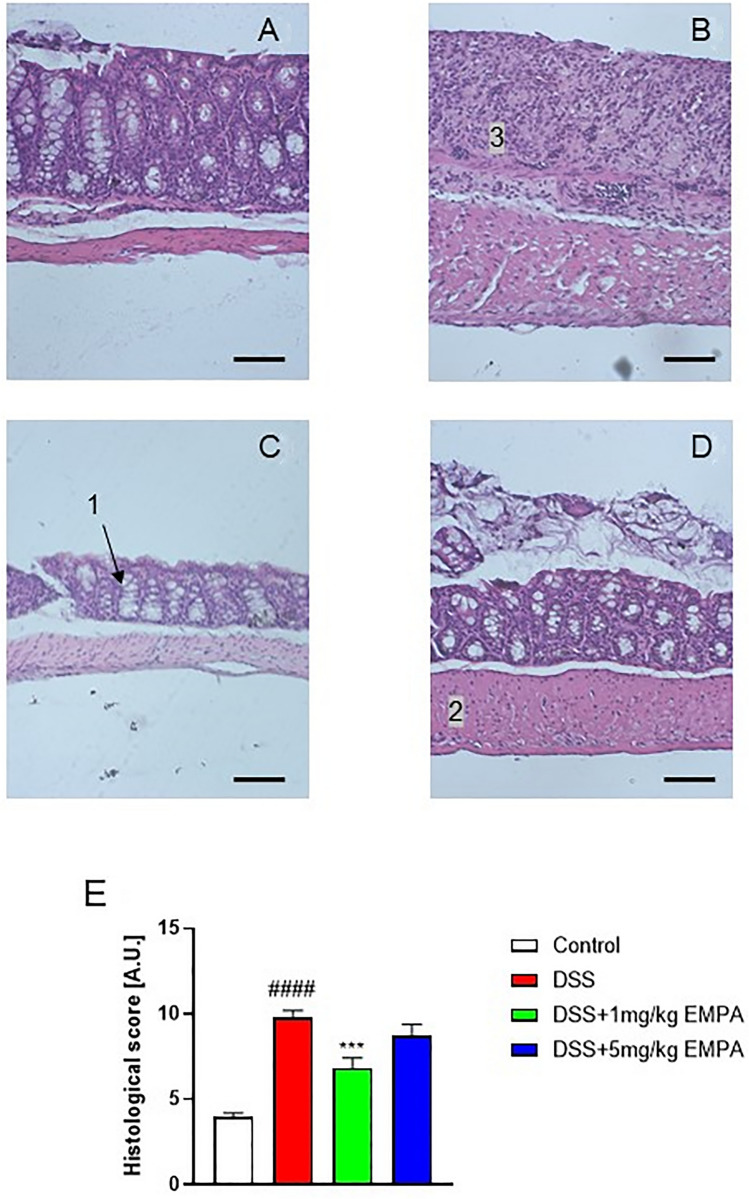
Representative micrographs of hematoxylin and eosin-stained sections of distal colon in acute DSS-induced colitis. Figure shows data for **A** Control, **B** DSS, **C** DSS + 1 mg/kg EMPA, **D** DSS + 5 mg/kg EMPA, and microscopic total damage score (**E**). Scale bar = 50 µm. Values are mean ± SEM, of 9–11 mice per group. Significance of differences between means: ^####^p < 0.0001 versus control group, ***p < 0.001 versus DSS-treated group. 1 Goblet cell, 2 mucosal layer, 3 cellular infiltration

Histological evaluation of mouse colon specimens supported the macroscopic observations (Fig. 7a–e). DSS treatment significantly increased the microscopic damage score, characterized by the thickening of muscular layer, increased immune cell in-filtration, loss of mucosal architecture, as well as altered crypts morphology (9.75 ± 0.45 for DSS vs 3.94 ± 0.25 for control, p < 0.0001) (Fig. 7a, b). EMPA at the dose of 1 mg/kg restored the microscopic architecture of the colon (6.81 ± 0.61, p = 0.0008) (Fig. 7c).

### Effect of EMPA on the chronic-relapsing model of DSS-induced colitis in mice

In further investigations, we evaluated the effect of EMPA in the model of chronic-relapsing DSS-induced colitis, which reflects exacerbations and remissions observed in IBD. In this model, colitis was induced by 3 cycles of 5 days treatment with 2% DSS in drinking water followed by 4 days of water without DSS. In the acute model of colitis, EMPA at the dose of 1 mg/kg displayed better anti-inflammatory properties than the dose of 5 mg/kg. Therefore, in the chronic-relapsing model we tested EMPA at the doses of 0.3 mg/kg and 1 mg/kg administered orally once daily from day 10 to day 27.

**Fig. 8:**
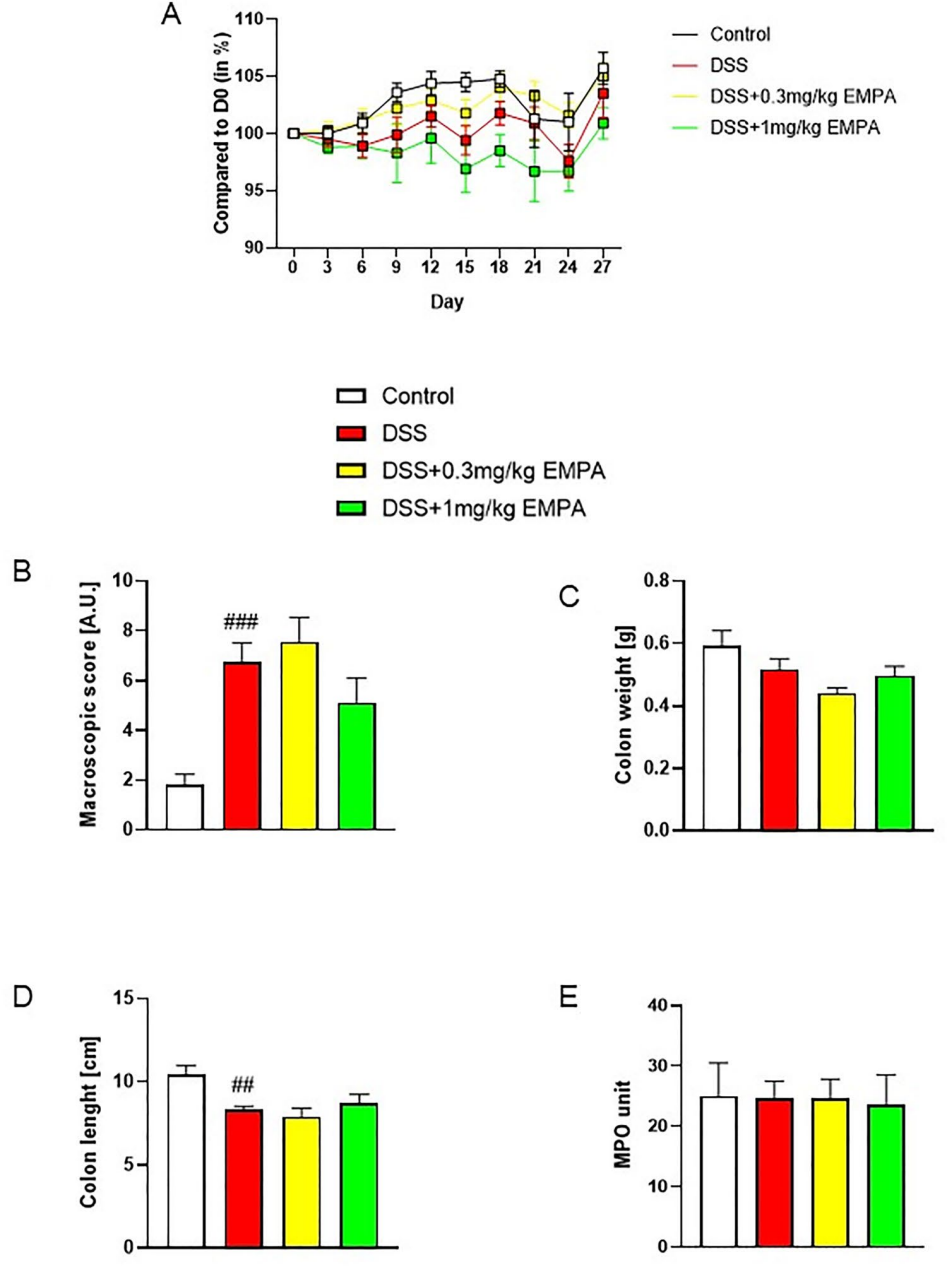
Effect of EMPA on parameters of chronic-relapsing inflammation in DSS-treated mice. EMPA was orally administered at two doses: 0.3 mg/kg or 1 mg/kg once daily (on days 10–27). Figure shows data for body weight (**A**), macroscopic score (**B**), colon weight (**C**), colon length (**D**), and MPO activity (**E**). Values are mean ± SEM, of 8–10 mice per group. Significance of differences between means: ^##^p < 0.01, ^###^p < 0.001 versus control group. A.U. arbitrary unit

**Fig. 9:**
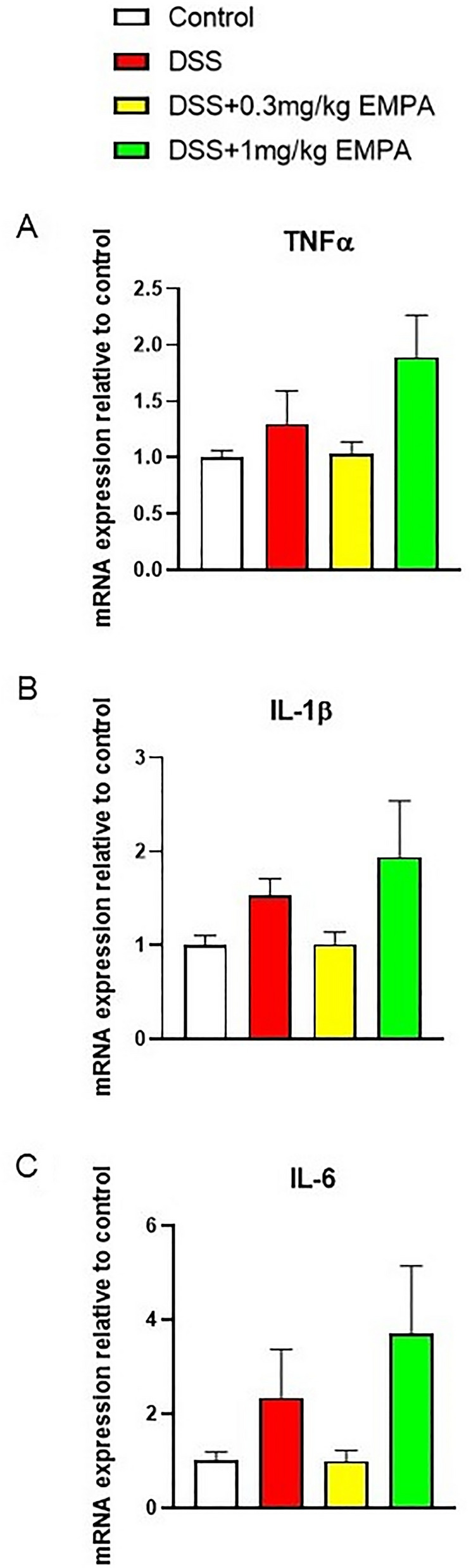
Effect of EMPA on pro-inflammatory cytokines gene expression in the colon of mice with acute DSS-induced colitis. EMPA was orally administered at two doses: 0.3 mg/kg or 1 mg/kg once daily (on days 10–27). Figure shows data for TNFα (**A**), IL-1β (**B**), and IL-6 (**C**) presented in relative units compared to control. Values are mean ± SEM, of 8–10 mice per group

**Fig. 10:**
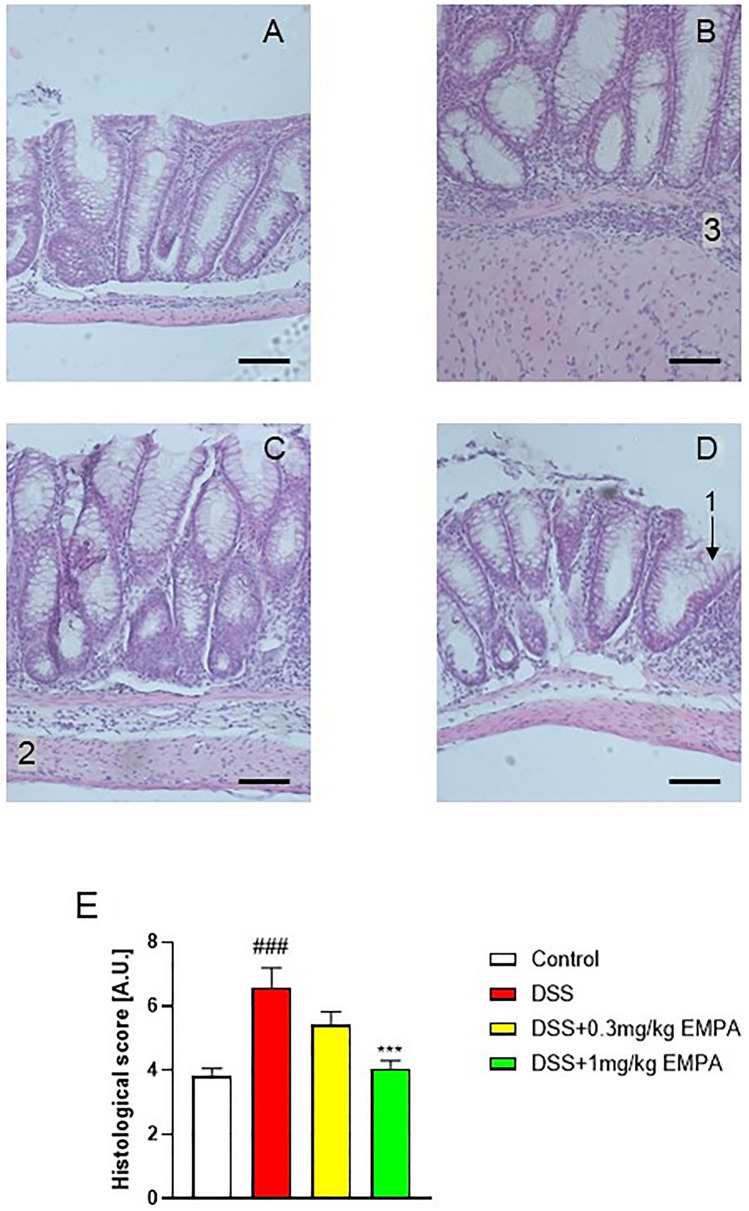
Representative micrographs of hematoxylin and eosin-stained sections of distal colon in chronic-relapsing DSS-induced colitis. Figure shows data for **A** Control, **B** DSS, **C** DSS + 0.3 mg/kg EMPA, **D** DSS + 1 mg/kg EMPA, and microscopic total damage score (**E**). Scale bar = 50 µm. Values are mean ± SEM, of 8–10 mice per group. Significance of differences between means: ^###^p < 0.001 versus control group, ***p < 0.001 versus DSS-treated group. 1 Goblet cell, 2 mucosal layer, 3 cellular infiltration

DSS treatment resulted in colonic inflammation, which was manifested by body weight loss, significantly increased macroscopic damage score and decreased colon length (Fig. 8a, b, d). EMPA did not alleviate macroscopic symptoms of colitis. Interestingly, contrary to acute model of colitis, the levels of MPO activity did not differ between the groups (Fig. 8e). Chronic administration of DSS up-regulated the expression of inflammatory cytokines, which was non-significantly influenced by the treatment with EMPA. The dose of 0.3 mg/kg EMPA inhibited this effect of DSS, whereas higher dose of 1 mg/kg increased the expression of cytokines (Fig. 9a–c). Similarly, to acute model of colitis, chronic-relapsing inflammation induced by DSS was characterized by increased microscopic damage score (6.58 ± 0.62 for DSS vs 3.83 ± 0.24 for control, p = 0.0001) (Fig. 10a, b). The histological changes were significantly reduced after treatment with 1 mg/kg of EMPA (4.06 ± 0.25, p = 0.0008) (Fig. 10d).

## Discussion

Large prevalence and recent significant increase in the incidence of IBD raise the need for the development of new, specific, and above all, effective therapeutics. For this purpose, in this study we focused our attention on gliflozins. This novel class of anti-diabetic agents inhibit SGLT-2 protein, which results in effective reduction of hyperglycemia. Moreover, plethora of studies showed that SGLT-2 inhibitors exhibit local and systemic anti-inflammatory properties (Kang et al. 2020). Hence, we tested the effect of three gliflozins; EMPA, DAPA and CANA in in vitro and in vivo models of intestinal inflammation.

Development of intestinal inflammation involves immune cells, especially macrophages, which are the main cells expressing iNOS. The expression of this enzyme is increased in response to cytokines as well as other activating stimuli, including microbiota playing key role in GI inflammation and further carcinogenesis (Ağagündüz et al. 2023). The process is characterized by upregulation of JAK/STAT and TLR-4/NF-κB signaling pathways, which lead to overproduction of NO. This molecule plays essential roles in intestinal physiology and pathology (Lanas 2008). In excessive amounts, NO exacerbates colonic inflammation, e.g. by the activation of innate immune system, induction of reactive nitric oxygen species (RNOS), and the release of intracellular cytotoxic agents. Studies showed that pharmacological agents, which suppress the pro-duction of NO, are also able to attenuate the symptoms of IBD (Kamalian et al. 2020). Our in vitro experiments revealed that EMPA and DAPA, but not CANA, suppressed the production of NO in LPS-treated murine RAW264.7 macrophages. In previous study on LPS-stimulated RAW264.7 cells, Lee et al. (2021) demonstrated that EMPA decreased the mRNA expression and protein level of iNOS as a result of JAK/STAT, NF-κB, and JNK pathways inhibition. Downregulation of TLR-4/NF-κB in LPS-stimulated macrophages was also observed for DAPA (Abdollahi et al. 2022). In another study, the inhibition of this pro-inflammatory cascade as well as the decrease in IL-1β level was observed in EMPA-treated RAW264.7 cells stimulated by oxLDL (Liu et al. 2021). Mentioned observations stay in line with our results.

We revealed that CANA did not attenuate NO synthesis in LPS-stimulated RAW264.7 cells. Interestingly, other studies on RAW264.7, THP-1, and human endothelial cells showed that CANA displays the strongest anti-inflammatory properties among all of tested gliflozins (Mancini et al. 2018; Uthman et al. 2021; Xu et al. 2018). It was suggested that the beneficial effect of CANA is partly due to the inhibition of intracellular glucose metabolism and this mechanism might be independent of SGLT-2 protein (Xu et al. 2018). We hypothesize that the divergence between the effects of CANA and other gliflozins on NO synthesis is caused by its lower selectivity compared to EMPA and DAPA which may cause off target effects. It is manifested by a relatively high affinity to SGLT-1, which is an isoform with wider tissue distribution than SGLT-2 (Sokolov et al. 2020). Several studies showed that SGLT-1 plays unclear roles in processes responsible for NO production. On the other hand, it is suggested that NO regulates SGLT-1 activity and not vice versa (Arthur et al. 2014; Palaniappan et al. 2020).

In order to explain the possible mechanisms underlying the anti-inflammatory properties of SGLT-2 inhibitors, we tested the effect of gliflozins on Caco-2 cells, which are widely used as a model of human intestinal epithelium. Contrary to our observations from RAW264.7 line, EMPA and DAPA did not influence the inflammatory response measured by IL-6 secretion after stimulation with pro-inflammatory cocktail. This may be caused by the low expression of SGLT-2 in Caco-2 cells and hence the lack of ability to mediate significant effects. It was showed that Caco-2 cells are characterized by having SGLT-1 as a major sodium-glucose co-transporter (Kipp et al. 2003).

In in vivo part of present study, we initially showed that EMPA partially alleviates acute DSS-induced colitis in mice. Even though we chose relatively low doses of orally administered EMPA, the dose of 1 mg/kg was more effective than 5 mg/kg. Such dose is approximate to a median effective dose of EMPA in mice (ED50 = 1.2 mg/kg), which was estimated by urinary glucose secretion (Michel et al. 2015). This observation suggests that partial inhibition of SGLT-2 might lead to better therapeutic effects than its saturation. We also found that orally administered 1 mg/kg of EMPA reduced macro- and microscopic colonic damage, as well as partially prevented from decreased expression of TJPs playing key role in intestinal barrier integrity. Moreover, EMPA attenuated biochemical inflammatory parameters including reduced activity of MPO, which is an oxidative enzyme produced primarily by neutrophils. This observation together with the results of in vitro experiments on macrophages suggest that gliflozins alleviate GI inflammation and should be considered in the treatment of patients with IBD and diabetes. We propose that anti-inflammatory properties of gliflozins result from their potent effects on innate immune cells. Moreover, we observed a stronger anti-inflammatory effect of empagliflozin in the acute colitis than in the chronic-relapsing model. This may be related to the different profiles of immune cells in the intestinal tissue depending on the degree of its inflammation. A study on trinitrobenzene sulfonic acid (TNBS)-induced colitis showed significantly increased innate immune responses in acute colitis as compared to colitis reactivated after remission. The colonic samples obtained from mice with acute colitis were characterized by higher macrophage infiltration and MPO activity, whereas infiltration of CD4 + lymphocytes was substantially lower than in reactivated, chronic colitis (Campaniello et al. 2017). In another mouse study, significant alterations in the number and distribution of lymphocytes were observed in the murine spleen, liver, and peripheral blood during the acute phase of DSS-induced colitis (Detel et al. 2016).Similarly, significant difference in the immune landscape was observed in a human study on UC individuals (Penrose et al. 2021).

The decrease in colonic level of MPO, and more generally an antioxidant effects of EMPA and DAPA were shown in two rat studies in DSS and AA-induced colitis, respectively (El-Rous et al. 2021; Youssef et al. 2021). In mentioned studies, anti-inflammatory properties of gliflozins were manifested by the reduction of disease activity indexes (DAIs), colon weight/length ratios, as well as macro- and microscopic damage scores. The use of EMPA and DAPA decreased expression of inflammatory cytokines. Additionally, EMPA prevented from DSS-induced changes in the expression of TJPs. Both studies indicated a possible mechanism underlying anti-inflammatory properties of SGLT-2 inhibitors. It involves phosphorylation of adenosine monophosphate (AMP)-activated protein kinase (AMPK) and subsequent downregulation of mammalian target of rapamycin (mTOR) and NOD-like receptor family pyrin domain-containing 3 (NLRP3). The activity of mTOR/NLRP3 inflammasome axis aggravates inflammation, which is a result of numerous processes including the promotion of differentiation of Th1 and Th17 immune cells.

Another study demonstrating anti-inflammatory effect of EMPA in AA-induced colitis in rats was performed by Zaghloul et al. (2022). Therapy with EMPA resulted in significant decrease of DAI, colon weight, weight/length ratio, and microscopic damage score. Considering biochemical parameters, EMPA significantly decreased serum lactate dehydrogenase (LDH) activity and CRP level, and restored colon redox balance biomarkers. Moreover, EMPA decreased colonic levels of TNFα, IL-1β, and IL-6, as well as increased the levels of TJP maintaining intestinal integrity. The study suggests another mechanism responsible for anti-inflammatory effects of SGLT-2 inhibitors. EMPA prevented from downregulation of silent information regulator-1 (SIRT-1) and following overactivity of NF-κB caused by AA. SIRT-1 is a deacetylase taking part in many processes, including regulation of inflammatory responses. There is a large evidence that optimal activity of SIRT-1 suppresses the hyperacetylation of NF-κB, which is an beneficial effect alleviating inflammation in IBD (Devi et al. 2020).

## Conclusions

Our study is another premise that SGLT-2 inhibitors act as anti-inflammatory agents independently from their hypoglycemic effects. Currently, the exact mechanisms explaining this beneficial property of SGLT-2 inhibitors are not fully understood. In the present study, we showed that such a mechanism may involve the influence of gliflozins on immune cells. We observed that EMPA and DAPA significantly attenuated inflammatory response in RAW264.7 macrophages in vitro. Moreover, we revealed anti-inflammatory activity of EMPA in in vivo model of acute colitis, in which the therapy resulted in the decrease of MPO activity. Our results suggest that the use of gliflozins may display some beneficial effects in the GI tract. Furthermore, SGLT-2 inhibitors may be considered in the development of new supplementary therapies for IBD patients. Future studies on this field are needed.

## Data Availability

The data presented in this study are available on request from the corresponding author.
